# The local immune response of mice after *Helicobacter suis* infection: strain differences and distinction with *Helicobacter pylori*

**DOI:** 10.1186/1297-9716-43-75

**Published:** 2012-10-29

**Authors:** Bram Flahou, Kim Van Deun, Frank Pasmans, Annemieke Smet, Jiri Volf, Ivan Rychlik, Richard Ducatelle, Freddy Haesebrouck

**Affiliations:** 1 Department of Pathology, Bacteriology and Avian Diseases, Faculty of Veterinary Medicine, Ghent University, Salisburylaan 133, Merelbeke, 9820, Belgium; 2 Veterinary Research Institute, Hudcova 70, Brno, 621 00, Czech Republic

## Abstract

*Helicobacter* (*H*.) *suis* colonizes the stomach of pigs and is the most prevalent gastric non-*H. pylori Helicobacter* species in humans. Limited information is available on host immune responses after infection with this agent and it is unknown if variation in virulence exists between different *H. suis* strains. Therefore, BALB/c and C57BL/6 mice were used to compare colonization ability and gene expression of various inflammatory cytokines, as determined by real-time PCR, after experimental infection with 9 different *H. suis* strains. All strains were able to persist in the stomach of mice, but the number of colonizing bacteria at 59 days post inoculation was higher in stomachs of C57BL/6 mice compared to BALB/c mice. All *H. suis* strains caused an upregulation of interleukin (IL)-17, which was more pronounced in BALB/c mice. This upregulation was inversely correlated with the number of colonizing bacteria. Most strains also caused an upregulation of regulatory IL-10, positively correlating with colonization in BALB/c mice. Only in C57BL/6 mice, upregulation of IL-1β was observed. Increased levels of IFN-γ mRNA were never detected, whereas most *H. suis* strains caused an upregulation of the Th2 signature cytokine IL-4, mainly in BALB/c mice. In conclusion, the genetic background of the murine strain has a clear impact on the colonization ability of different *H. suis* strains and the immune response they evoke. A predominant Th17 response was observed, accompanied by a mild Th2 response, which is different from the Th17/Th1 response evoked by *H. pylori* infection.

## Introduction

The gastric mucosa of pigs is often colonized by *H. suis*[1-5], a large spiral-shaped bacterium which is also the most prevalent non-*H. pylori Helicobacter* species in humans [6,7]. In pigs, *H. suis* infection has been associated with gastritis, ulceration of the non-glandular part of the stomach and a decreased daily weight gain [7-11]. In humans, *H. suis* infection may cause gastritis, peptic ulcer disease and gastric mucosa-associated lymphoid tissue (MALT) lymphoma [12-14]. Interestingly, the risk of developing MALT lymphoma is higher after infection with non-*H. pylori Helicobacter* species than after infection with *H. pylori*[12,15].

For *H. pylori,* it is well known that different strains may vary in virulence and in the host immune response they evoke [16,17]. This host immune response plays an important role in induction and evolution of gastric lesions and influences colonization of the gastric mucosa by *H. pylori*[16]*.* For *H. suis,* however, nothing is known about possible differences in virulence between bacterial strains. Some studies have attempted to characterize the evoked immune response in the mouse, an animal species which has most often been used to model gastric *Helicobacter* infection. However, because of the fastidious nature of this micro-organism, these experimental infection studies were carried out using impure mucus or homogenized gastric tissue from infected mice, pigs or non-human primates, thus hampering interpretation of these results [18-20].

Besides the possible difference in virulence and immune response evoked by different bacterial strains, also the choice of the murine strain is important. Inbred mouse strains can vary greatly in colonization susceptibility and their immune response towards *Helicobacter* infections [21-23]. C57BL/6 mice have been described genetically as Th1 responders, whereas BALB/c mice are considered predominant Th2 responders [24,25]. Therefore, in the present study, BALB/c and C57BL/6 mice were used to compare colonization ability and host responses after inoculation with nine different *H. suis* strains. The local immune response was investigated and compared to that evoked by infection with *H. pylori*.

## Material and methods

### Bacterial strains

*H. suis* strains HS1, HS2, HS3, HS4, HS5, HS6, HS7, HS8, HS9 were isolated from the stomachs of pigs belonging to different herds. Results from Multilocus Sequence Typing have shown that these isolates indeed correspond to 9 different strains (Liang et al., unpublished observations). The strains were grown for 72 h as described previously [23] under microaerobic conditions (37°C; 85% N_2_, 10% CO_2_, 5% O_2_) on biphasic Brucella (BD, Franklin Lakes, NJ, USA) culture plates supplemented with 20% fetal calf serum (HyClone, Logan, UT, USA), 5 mg/L amphotericin B (Fungizone; Bristol-Myers Squibb, Epernon, France), *Campylobacter* selective supplement (Skirrow; Oxoid, Basingstoke, UK) and Vitox supplement (Oxoid). In addition, the pH of the agar and overlying broth was adjusted to 5.

The mouse-adapted *H. pylori* strain SS1 and its parental strain pMSS1 were grown for 48 h at 37°C under microaerobic conditions on Columbia agar plates containing 5% sheep blood (Oxoid, Basingstoke, UK). Subsequently, colonies were picked up and cultured in Brucella broth (without pH adjustment), supplemented with Skirrow and Vitox, on a rotational shaker under microaerobic conditions (16 h, 150 rpm).

### Animals and experimental design

Seven-week-old, female specific-pathogen-free BALB/c and C57BL/6 mice, free of *Helicobacter* spp., were purchased from Harlan NL (Horst, The Netherlands). Both for BALB/c and C57BL/6 mice, 9 groups consisting of 5 mice each were inoculated with different *H. suis* strains twice with a 48 h interval. Under brief isoflurane anaesthesia and using a ball-tipped gavage needle, 250 μL Brucella broth (pH5) containing 7 × 10^7^ bacteria of the different *H. suis* strains was administered intragastrically. BALB/c and C57BL/6 control groups consisted of 5 mice receiving an equal volume of Brucella broth with a pH of 5. In order to compare the *H.* suis-induced T helper cell response to that evoked by *H. pylori* infection, BALB/c and C57BL/6 mice were likewise inoculated twice with 250 μL Brucella broth (without adjusted pH) containing 7 × 10^7^ bacteria of *H. pylori* strains SS1 and pMSS1 (6 animals of each mouse strain for each *H. pylori* strain). Six Brucella broth (without adjusted pH)-inoculated BALB/c and C57BL/6 mice were included as controls. Fifty-nine days after the first inoculation, all mice were euthanized and the stomachs were removed for further processing. All animal procedures were approved by the Ethical committee of the Faculty of Veterinary Medicine, Ghent University (EC2009/055 and EC2012/086).

### Quantification of colonizing *H. suis* and *H. pylori* bacteria

The number of colonizing *H. suis* bacteria per mg gastric tissue was determined by quantitative RT-PCR. In brief, a 1146 bp segment of the *ureB* gene served as standard (primers: UreaseB forward 5^′^- CGG GAT TGA TAC CCA CAT TC-3^′^; reverse 5^′^- ATG CCG TTT TCA TAA GCC AC-3^′^). The copy number concentration was calculated based on the length of the amplicon and the mass concentration. The standard consisted of 10-fold dilutions starting at 10^7^ PCR amplicons for each 10 μL of reaction mixture. For enumeration of *H. suis* in stomach samples, stomachs were homogenized in 1 mL TRI Reagent RT (MRC, Brunschwig Chemie, Amsterdam, The Netherlands) using a MagNA Lyser (Roche Applied Science, Mannheim, Germany). After centrifugation of the obtained homogenate, the interphase and organic phase were stored at −20°C for DNA isolation for enumeration of *H. suis* bacteria. One μL of this extracted DNA was used as template in 10 μL reactions, further containing 5 μL iQ SYBR® Green Supermix and 5 pmol of both primers, located within the 1146 bp fragment, to yield a 218 bp PCR product (Sense primer: 5^′^-TTA CCA AAA ACA CCG AAG CC-3^′^, antisense primer: 5^′^-CCA AGT GCG GGT AAT CAC TT-3^′^; annealing temperature: 60°C). Both standards and samples were run in duplicate and the average values were used for quantification of *H. suis*.

A similar DNA extraction method and RT-PCR was applied for quantification of *H. pylori*. For generation of the standard, part of the *ureAB* gene cluster from *H. pylori* strain SS1 was amplified using consensus primers U430F and U1735R, as described by O’Rourke et al. [26]. For amplification of an internal 217 bp fragment, the following primers were designed: 5^′^-AAA GAG CGT GGT TTT CAT GGC G-3^′^ and 5^′^-GGG TTT TAC CGC CAC CGA ATT TAA-3^′^.

### Cytokine expression

Quantitative Real-Time PCR (RT PCR) was used to examine cytokine expression in stomach tissue. After stomach homogenization and centrifugation of the obtained homogenate as described above, the interphase and organic phase were stored at −20°C for DNA isolation for enumeration of *H. suis* or *H. pylori* bacteria (see above). Total RNA was extracted from the upper aqueous phase using the RNeasy mini kit (Qiagen, Venlo, The Netherlands) according to the manufacturer’s guidelines. RNA (1 μg) was reverse transcribed to cDNA with the iScript cDNA synthesis kit using a mix of oligo(dT) and random hexamer primers (Biorad, Nazareth, Belgium), and mRNA expression levels of various cytokines (IL-1β, IL-2, IL-4, IL-5, IL-6, IFN-γ, IL-10, IL-12b, IL-17, TNF-α and MIP-2 for *H. suis*-infected mice; IL-4, IL-10, IL-17, IFN-γ for *H. pylori*-infected mice; primer sequences are shown in Table 1) was quantified using SYBR Green based RT PCR with iQ SYBR® Green Supermix and performed on a CFX96 RT PCR System with a C1000 Thermal Cycler (Biorad). The housekeeping genes *H2afz*, *PPIA* and *HPRT* were shown to have a stable mRNA expression in all samples tested (data not shown) and were included as references. Reactions were performed in 10 μL volumes containing 5 pmol of both forward and reverse primers, 5 μL iQ SYBR® Green Supermix and 1 μL cDNA. The thermal cycle program consisted of 95°C for 15 min, followed by 40 cycles of denaturation at 95°C for 20 s and annealing/extension at 60°C for 30 s. The threshold cycle values (Ct) were first normalized to the geometric means of the reference genes and the normalized mRNA levels were calculated according to 2^−ΔΔCt^ method for each individual animal [27].

**Table 1:** List of genes and sequences of the primers used for RT-PCR gene expression analysis

**Gene**	**Primer**	**Primer sequence**
*HPRT*	sense	5^′^- CAG GCC AGA CTT TGT TGG AT-3^′^
	antisense	5^′^-TTG CGC TCA TCT TAG GCT TT-3^′^
*PPIA*	sense	5^′^-AGC ATA CAG GTC CTG GCA TC-3^′^
	antisense	5^′^-TTC ACC TTC CCA AAG ACC AC-3^′^
*H2afz*	sense	5^′^-CGT ATC ACC CCT CGT CAC TT-3^′^
	antisense	5^′^-TCA GCG ATT TGT GGA TGT GT-3^′^
*IL-1β*	sense	5^′^- GGG CCT CAA AGG AAA GAA TC-3^′^
	antisense	5^′^-TAC CAG TTG GGG AAC TCT GC-3^′^
*IL-2*	sense	5^′^-TTT CAA TTG GAA GAT GCT GAG A-3^′^
	antisense	5^′^-AGG GCT TGT TGA GAT GAT GC-3^′^
*IL-4*	sense	5^′^-ACT CTT TCG GGC TTT TCG AT-3^′^
	antisense	5^′^-AAA AAT TCA TAA GTT AAA GCA TGG TG-3^′^
*IL-5*	sense	5^′^-GTG GGG GTA CTG TGG AAA TG-3^′^
	antisense	5^′^-TCC TCG CCA CAC TTC TCT TT-3^′^
*IL-6*	sense	5^′^-CAA AGC CAG AGT CCT TCA GAG-3^′^
	antisense	5^′^-GCC ACT CCT TCT GTG ACT CC-3^′^
*IFN-γ*	sense	5^′^- GCG TCA TTG AAT CAC ACC TG-3^′^
	antisense	5^′^-TGA GCT CAT TGA ATG CTT GG-3^′^
*IL-10*	sense	5^′^-ATC GAT TTC TCC CCT GTG AA-3^′^
	antisense	5^′^-CAC ACT GCA GGT GTT TTA GCT T-3^′^
*IL-12b*	sense	5^′^-TAA CCA GAA AGG TGC GTT CC-3’
	antisense	5^′^-CTT TCC AAC GTT GCA TCC TA-3^′^
*IL-17*	sense	5^′^-TTT AAC TCC CTT GGC GCA AAA-3^′^
	antisense	5^′^-CTT TCC CTC CGC ATT GAC AC-3^′^
*TNF-α*	sense	5^′^-CAA ATG GCC TCC CTC TCA T-3^′^
	antisense	5^′^-GGT TGT CTT TGA GAT CCA TGC-3^′^
*MIP-2*	sense	5^′^-AAA GTT TGC CTT GAC CCT GA-3^′^
	antisense	5^′^-TCC AGG TCA GTT AGC CTT GC-3^′^

### Data analysis

For relative gene expression, assessment of significance was done with SPSS19 software, using a non-parametric Mann–Whitney *U* test, or with the Relative Expression Software Tool (REST 2009 V2.0.13). A Bonferroni correction was applied to investigate differences between groups regarding gene expression. A non-parametric Kruskal-Wallis and post-hoc Mann–Whitney *U* test with Bonferroni correction (SPSS 16) was used to investigate colonization differences. For correlation between different variables, Spearman’s rho correlation coefficients were calculated. Data is expressed as the mean ± standard deviation. In general, *p* values < 0.05 were considered statistically significant.

## Results

### *H. suis* colonizes C57BL/6 mice to a higher degree compared to BALB/c mice

**Figure 1:**
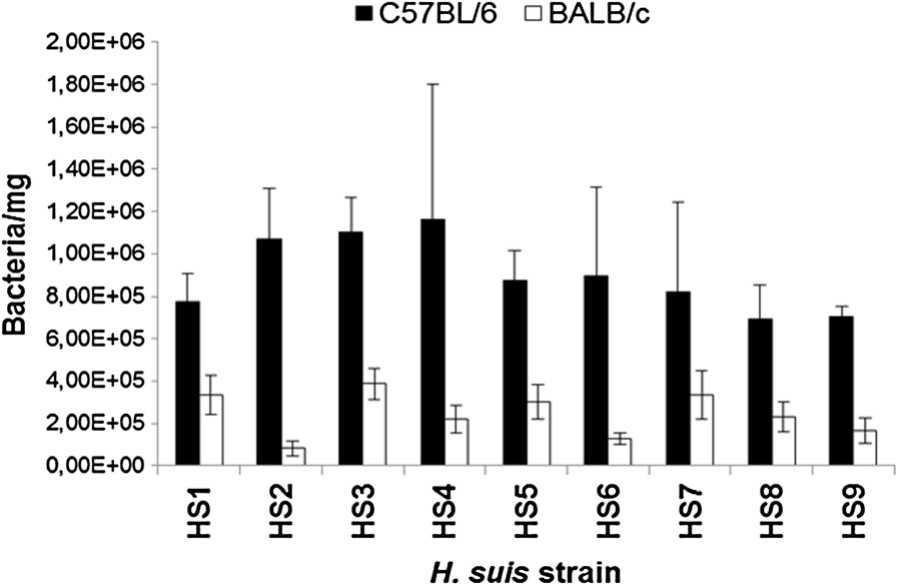
**Number of colonizing *H. suis *bacteria.** Shown is the average number of *H. suis* bacteria/milligram tissue in the stomach of BALB/c and C57BL/6 mice 59 days after experimental infection.

Fifty nine days after the first inoculation, colonizing *H. suis* bacteria were enumerated. All mice, except the controls, were colonized by *H. suis* bacteria, regardless of the mouse strain or *H. suis* strain involved. The detection limit was 180 bacteria per mg tissue. Generally, BALB/c mice showed 3.5 times lower bacterial numbers in gastric tissue compared to C57BL/6 mice (*p* < 0.001). The mean number of bacteria/milligram tissue was 2.40 (± 0.27) × 10^5^ bacteria for BALB/c mice (*n* = 44), compared to 8.37 (± 1.02) × 10^5^ bacteria for C57BL/6 mice (*n* = 44). The *H. suis* strains were not different from each other in their capacity to colonize mice. Data is summarized in Figure 1.

In the *H. pylori*-infected groups, lower colonization rates were observed compared to *H. suis*: 1.63 (± 1.16) × 10^1^ bacteria/mg tissue for pMSS1-infected C57BL/6 mice; 4.65 (± 4.64) × 10^2^ bacteria/mg tissue for SS1-infected C57BL/6 mice; 2.95 (± 2.48) × 10^3^ bacteria/mg for SS1-infected BALB/c mice. Strain pMSS1 could not be demonstrated in the stomach of BALB/c mice 59 days after inoculation.

### All 9 *H. suis* strains induce a predominant Th17 response in both BALB/c and C57BL/6 mice

**Figure 2:**
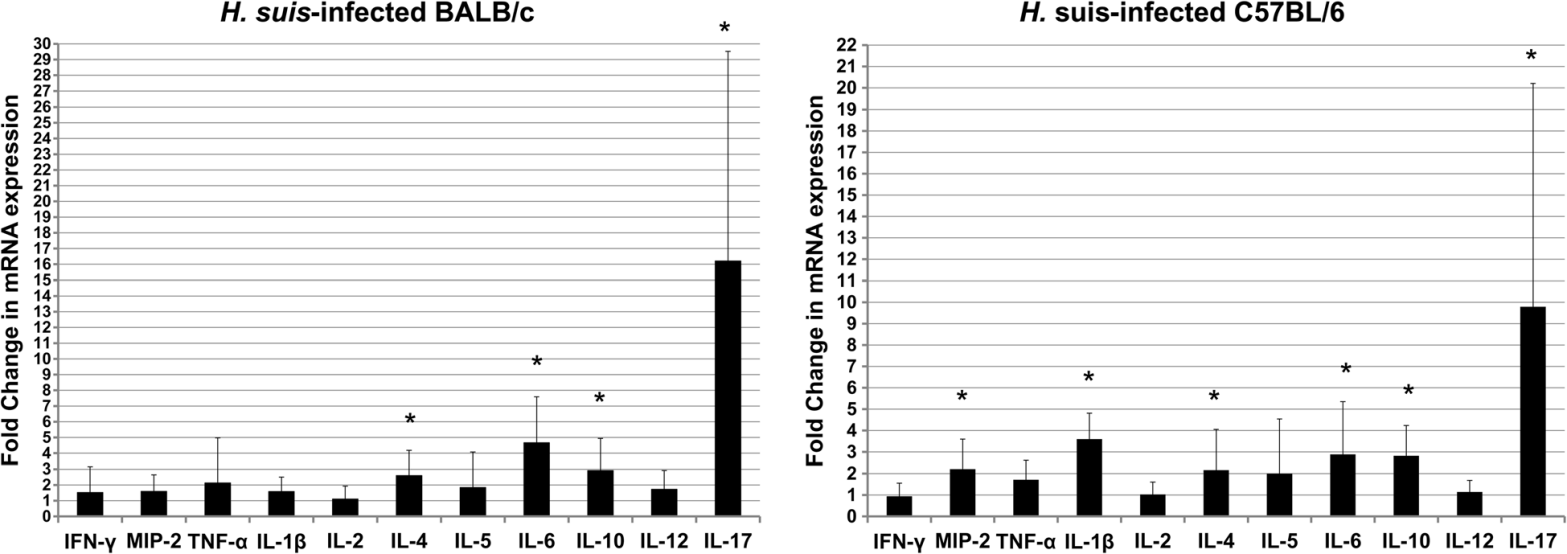
**General cytokine expression profile after experimental *H. suis *infection in BALB/c and C57BL/6 mice.** Shown are the mean fold changes in mRNA expression of indicated cytokines in *H. suis*-infected BALB/c and C57BL/6 mice. The mean fold change in the relevant uninfected control groups is equal to 1. An * indicates a statistically significant difference compared to uninfected control mice (*p* < 0.05).

**Figure 3:**
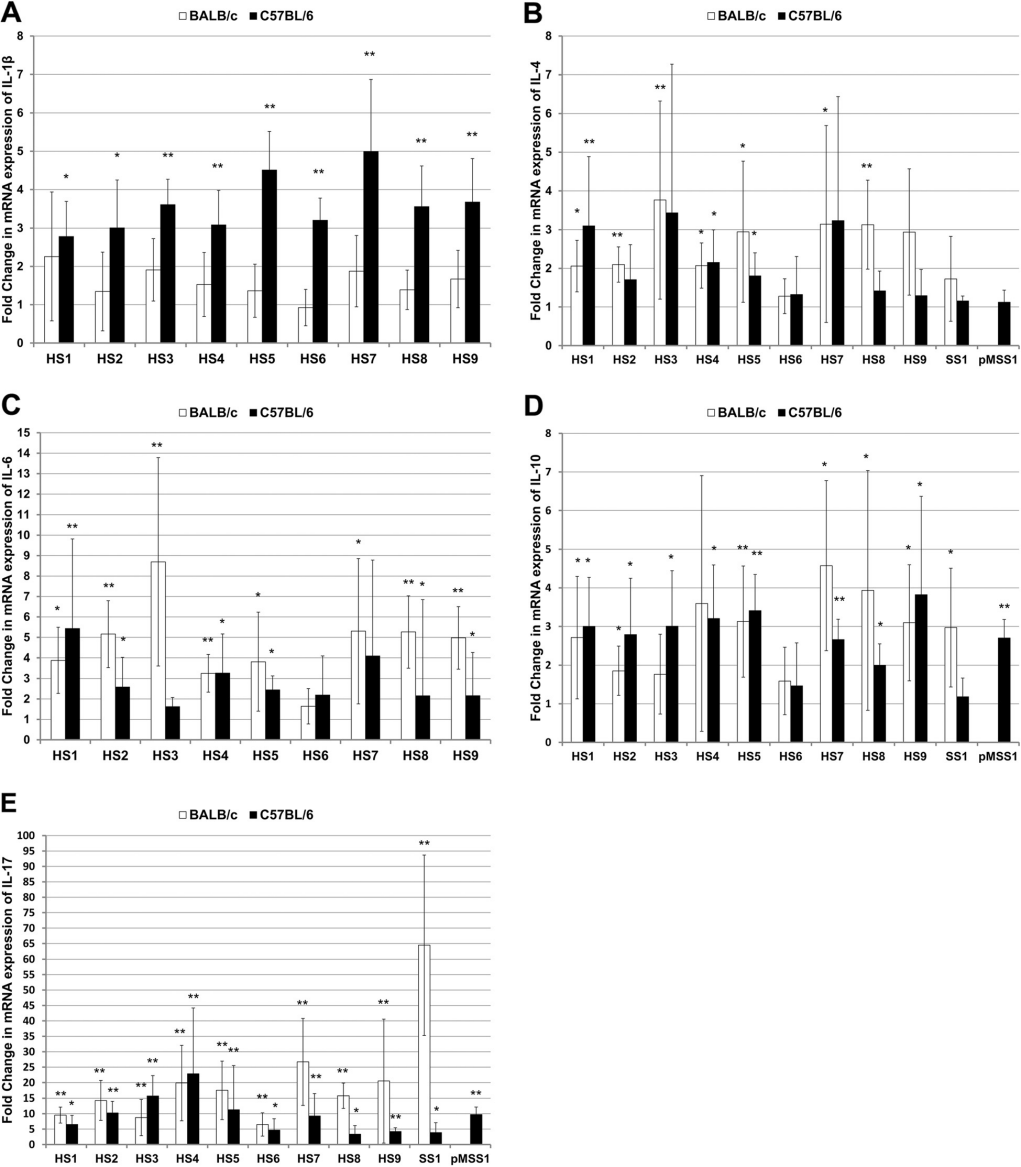
**Cytokine expression profiles for each group of animals infected with *H. suis *strains HS1-9 and *H. pylori *strains SS1 or pMSS1.** Shown are the mean fold changes of mRNA expression in HS1-HS9- and SS1- or pMSS1-infected BALB/c and C57BL/6 mice for IL-1β (**A**), IL-4 (**B**), IL-6 (**C**), IL-10 (**D**) and IL-17 (**E**). The mean fold change in the relevant uninfected control groups is equal to 1. An * indicates a significant upregulation of mRNA expression compared to uninfected control mice (*p* < 0.05). An ** indicates a significant upregulation of mRNA expression compared to uninfected control mice (*p* < 0.0056, i.e. the new cut-off *p* value obtained after Bonferroni correction for multiple comparisons).

To investigate the immune response of BALB/c and C57BL/6 to different *H. suis* strains, cytokine expression was evaluated. Results are shown in Figures 2 and 3.

IL-2 is a pleiotropic cytokine playing an important role in proliferation of lymphocytes and modulation of effector (T) cell differentiation, including inhibition of Th17 development. For this cytokine, no difference in expression was observed between *H. suis*-infected and non-infected control mice. Compared to control animals, with fold change mRNA expression levels equal to 1.0, the mean relative expression was 1.11 ± 0.82 and 1.03 ± 0.58 fold for *H.* suis-infected BALB/c and C57BL/6 mice, respectively.

Also for IFN-γ, a signature Th1 marker, no differences in expression were observed between *H. suis*-infected and control mice. Compared to control animals, the mean relative expression was 1.32 ± 1.29 and 0.94 ± 0.61 fold for *H. suis*-infected BALB/c and C57BL/6 mice, respectively. This striking absence of a Th1 response was confirmed by the lack of proinflammatory IL-12b upregulation. In contrast, a statistically significant upregulation of IFN-γ expression was observed for *H. pylori* pMSS1-infected C57BL/6 mice (2.4 ± 0.61 fold; *p* < 0.05). *H. pylori* strain SS1 did not cause a significant upregulation of IFN-γ expression, neither in BALB/c mice (2.67 ± 2.10 fold; *p* = 0.151), nor in C57BL/6 mice (1.0 ± 0.15 fold; *p* = 0.631).

In general, compared to uninfected animals, expression of IL-4, a Th2 signature cytokine, was upregulated in *H. suis*-infected mice from both strains (2.60 ± 1.58 fold for BALB/c mice, *p* < 0.01; 2.16 ± 1.88 fold for C57BL/6 mice, *p* < 0.05). This increased mRNA expression level was not observed for all *H. suis* strains: 3 and 1 out of 9 *H. suis* strains caused an upregulation of IL-4 in BALB/c and C57BL/6 mice, respectively, as shown by a *p* value below 0.0056, the new cut-off *p* value obtained after Bonferroni correction. However, *p* values for an additional 4 and 2 strains were lower than 0.05, further confirming this trend towards a Th2 response. In *H. pylori* SS1- or pMSS1-infected mice, no upregulation of IL-4 was observed.

No differences in IL-5 expression were detected when comparing uninfected and *H. suis*-infected animals.

**Figure 4:**
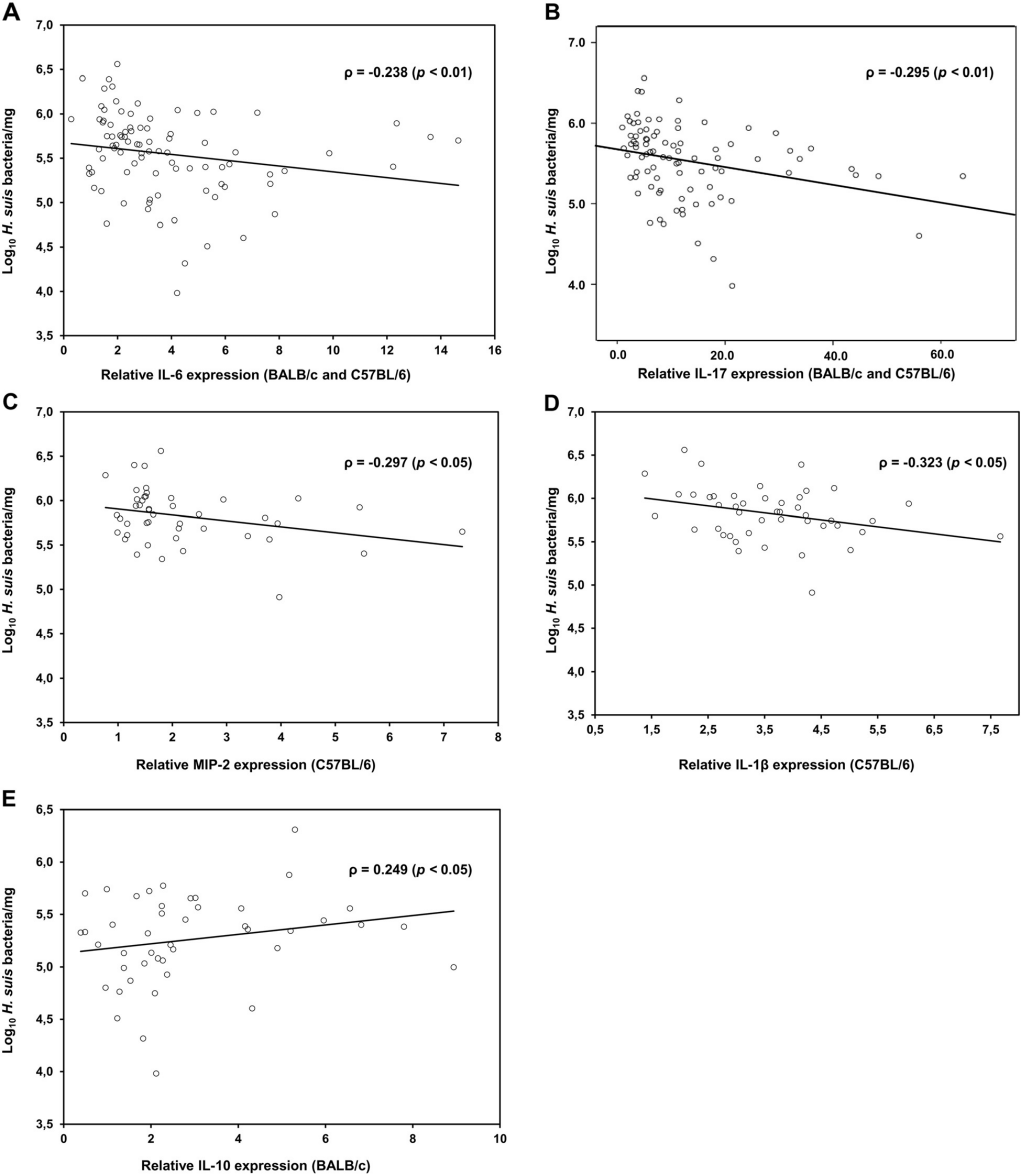
**Correlation between cytokine expression and *H. suis *colonization.** Shown are the correlation analyses between IL-6 (**A**), IL-17 (**B**), MIP-2 (**C**), IL-1β (**D**) and IL-10 (**E**) mRNA expression levels and the number of colonizing *H. suis* bacteria in stomachs of indicated mouse strains (BALB/c and C57BL/6). Correlation was measured by Spearman’s Rho (ρ).

Compared to uninfected animals, expression of IL-6, involved in Th2 and Th17 differentiation, was upregulated in *H. suis*-infected animals from both mouse strains (mean fold change: 4.67 ± 2.92 for BALB/c mice; 2.89 ± 2.45 for C57BL/6 mice) (*p* < 0.01). This increased mRNA expression level was observed for 6 out of 9 strains in C57BL/6 mice and even for 8 out of 9 *H. suis* strains in BALB/c mice when ignoring Bonferroni correction. A significant inverse correlation was observed between IL-6 expression and *H. suis* colonization (Figure 4a; *p* < 0.01; *ρ* = −0.238).

In contrast to the absent upregulation of Th1 cytokines and the mild upregulation of Th2 markers, a clear Th17 response was observed in both mouse strains for all *H. suis* strains, as illustrated by a marked IL-17 upregulation (*p* < 0.01). The mean upregulation of IL-17 in BALB/c mice was 16.21 ± 13.32 fold and 9.79 ± 10.41 fold in C57BL/6 mice. A significant inverse correlation was observed between IL-17 expression and colonization (*p* < 0.01; *ρ* = −0.295) (Figure 4b). Also in *H. pylori*-infected mice, a significant upregulation of IL-17 expression (up to 65 fold in SS1-infected BALB/c mice) could be observed.

### C57BL/6 mice but not BALB/c mice upregulate IL-1β expression as a response to *H. suis* colonization

Three important proinflammatory mediators of the innate immune system, TNF-α, MIP-2 and IL-1β, were also investigated (Figures 2 and 3). IL-1β was significantly upregulated only in the C57BL/6 strain: 7 out of 9 *H. suis* strains caused an upregulation (*p* < 0.0056) and overall average expression was 3.60 ± 1.22 fold. Generally, only in C57BL/6 mice, a slight upregulation of MIP-2 was observed (2.21 ± 1.39 fold) (*p* < 0.05). For MIP-2 and IL-1β, a significant inverse correlation was observed between increased expression and *H. suis* colonization in C57BL/6 mice (*ρ* = −0.297, *p* < 0.05; *ρ* = −0.323, *p* < 0.05; Figures 4c and 4d). No changes in mRNA expression of TNF-α were detected.

### Anti-inflammatory IL-10 is upregulated both in BALB/c and C57BL/6 mice during colonization by *H. suis*

As presented in Figures 2 and 3, increased expression of IL-10 was observed in both mouse strains inoculated with *H. suis* (2.92 ± 2.02 fold for BALB/c mice; 2.82 ± 1.41 fold for C57BL/6 mice) (*p* < 0.05). Similar levels of IL-10 upregulation were observed in *H. pylori*-infected mice of both strains.

When taking all *H.* suis-infected animals from both mouse strains into account, IL- 10 expression did not correlate with colonization (*p* > 0.05). This was, however, the case when analysis was restricted to *H. suis*-infected BALB/c mice (*ρ* = 0.249, *p* < 0.05; Figure 4e).

## Discussion

In the present study, all 9 *H. suis* strains were able to persist in the stomach of 2 different mouse strains at relatively high colonization levels compared to *H. pylori*. Most likely, colonization with *H. suis* is achieved more easily compared to *H. pylori*, which often requires prior adaptation to mice. This was underlined by the higher colonization rates observed for the mouse-adapted *H. pylori* SS1 strain, compared to the parental pre-mouse SS1 (pMSS1) strain [28,29]. The ease by which mice become infected with *H. suis* warrants further research on the role of mice in the epidemiology of *H. suis* infections in pig herds.

In general, *H.* suis-infected BALB/c mice showed lower colonization rates compared to C57BL/6 mice. A discrepancy between the colonization of BALB/c and C57BL/6 mice has also been described for other gastric helicobacters such as *H. felis* and *H. pylori*[21,30,31]. The higher inflammatory response, as observed histologically in the stomach of BALB/c mice infected with *H. suis*[23], may create a hostile and unfavourable environment for the bacterium, as has been suggested previously [32]. One possible factor contributing to the lower degree of *H. suis* colonization observed in BALB/c mice, could be the more pronounced Th17 response in this mouse strain. Although in both mouse strains an infection with *H. suis* induced an upregulation of IL-17 mRNA, the response was significantly higher in BALB/c mice. A clear inverse correlation between IL-17 mRNA and IL-6 mRNA (promoting Th17) expression levels on the one hand and *H. suis* colonization on the other hand was observed in this study, which largely corresponds to what has been described for *H. pylori*. Indeed, most studies reveal a negative correlation between a fully functional Th17 response and *H. pylori* colonization [33,34], although some authors suggest the opposite [35].

Besides a Th17 response, *H. pylori* has been shown to upregulate the Th1 response both in humans and in mouse models [35-37]. This was indeed confirmed in this study with *H. pylori* strain pMSS1, although levels of IFN-γ upregulation were increased only mildly. Interestingly, no increased expression of IFN-γ, a signature Th1 cytokine, could be observed in *H. suis*-infected animals in the present study, neither in BALB/c mice nor in Th1-prone C57BL/6 mice [24,25]. In contrast, most *H. suis* strains caused an upregulation of the Th2 signature cytokine IL-4, which was most pronounced in BALB/c mice. A similar upregulation was absent in *H. pylori*-infected mice, clearly highlighting the differences in the immune response elicited in mice infected with *H. pylori* (Th17/Th1) compared to mice infected with *H. suis* (Th17/Th2). Moreover, the results obtained in this study correspond to the histological changes observed in mice and Mongolian gerbils infected for up to 8 months with *H. suis* strain 5 [23]. In this study, only in BALB/c mice infected with HS5 for 8 months, an increased B cell accumulation was observed, further underlining the involvement of a Th2-polarized response [25]. Interestingly, a Th2 response, rather than a Th1-predominant response, has been associated with the development of low-grade B cell MALT lymphoma [38,39], which has indeed been associated with gastric non-*H. pylori Helicobacter* infection, including *H. suis*[12].

As discussed above, the overall inflammatory response, including the Th17 response, is stronger in *H. suis*-infected BALB/c mice, compared to C57BL/6 mice. IL-10, often produced by regulatory T cells (Treg’s), is a suppressive cytokine that acts through downregulation of several pro-inflammatory cytokines and the resulting decrease of inflammatory cell recruitment [40]. For *H. pylori*, it has been shown that infection suppresses the effective induction of *H. pylori*–specific Th17 immunity through the induction of a Treg response [34]. A positive correlation between expression of IL-10, a suppressive cytokine, and *H. suis* colonization could be observed only in BALB/c mice, although similar levels of IL-10 expression were observed in *H. suis*-infected mice from both strains. So most likely, other mechanisms, such as the difference in IL-6 expression, are involved since this cytokine promotes a Th17, as well as a Th2 response, both of which are more pronounced in BALB/c mice [34,41].

In a previous experimental infection study in mice with *H. suis* strain HS5, we observed only a mild increase of macrophage infiltration in the corpus of the stomach at 21 and 63 days after infection, but not at 8 months post infection [23]. In the present study, no significant upregulation of TNF-α and IL-12 expression was observed in animals infected with *H. suis* for 59 days. Possibly, the absent upregulation of these macrophage-secreted cytokines reflects the return of macrophage infiltration to baseline levels after a possible mild increase during the initial weeks of *H. suis* infection.

Only in C57BL/6 mice, an IL-1β upregulation was observed in *H. suis*-infected animals. It has been shown that this pro-inflammatory cytokine also plays an important role in the inhibition of gastric acid secretion and the development of severe hyperplastic and dysplastic glandular changes [42,43]. However, previous experiments showed that these lesions do not develop in C57BL/6 nor BALB/c mice after long-term (8 months) infection with *H. suis*[23]. Instead, lymphoid tissue lesions develop both in mice and Mongolian gerbils, which contrasts to the predominant metaplastic/dysplastic changes observed during long-term *H. pylori* infection [23,44].

Although all *H. suis* strains included in this study induced a similar immune response, some remarkable differences could be observed. Most *H. suis* strains caused no significant upregulation of IL-4 in C57BL/6 mice, although this was the case for HS1. In addition, HS1 induced the strongest upregulation in this mouse strain of IL-6, involved in Th17 and Th2 differentiation. On the other hand, only one strain, HS6, showed a complete lack of IL-4, IL-6 and IL-10 upregulation in both mouse strains. This clearly shows that mild strain differences exist in the immune response evoked by *H. suis* in the murine host. Although this is most likely also the case in infected humans and pigs, this remains to be investigated.

For *H. pylori,* differences in the host response evoked by different strains have been associated with the presence, absence or functionality of several virulence-associated genes, for instance those belonging to the cytotoxin-associated genes pathogenicity island (*cag*PAI) and the vacuolation cytotoxin A encoding gene *vacA*[45-47]. *H. suis* lacks most of the genes of the *cag*PAI and does not produce a functional vacuolating cytotoxin A [48]. One of its main virulence factors, capable of modulating lymphocyte function through its effects on lymphocyte proliferation and cytokine secretion, is the gamma-glutamyl transpeptidase [49]. It remains to be determined if differences in production of this enzyme by different *H. suis* strains, as has indeed been observed in vitro [50], play a role in the mild variation of the immune response described in the present study.

In conclusion, all 9 *H. suis* strains were capable of colonizing mice, but the numbers of *H. suis* bacteria were lower in the stomach of BALB/c mice. Although differences between *H. suis* strains were observed, colonization generally caused a predominant Th17 response, mainly in BALB/c mice, accompanied by a less pronounced Th2 response for most *H. suis* strains. This contrasts with the immune response induced by *H. pylori* infection, characterized by a Th17/Th1 response and the absence of a Th2 response. Despite a clear immune response evoked in the murine host, infection persisted in all *H. suis*-inoculated animals.

## Competing interests

The authors declare that they have no competing interests.

## Author’s contributions

BF participated in the design of the study, carried out the experiments, analysed the data and drafted the manuscript. KVD participated in the design of the study, carried out the experiments, analysed the data and participated in drafting the manuscript. FP participated in the design of the study and drafting of the manuscript. AS helped performing the experiments. JV and IR participated in preliminary data acquisition. RD and FH coordinated the study and participated in the design of the study and drafting of the manuscript. All authors read and approved the final manuscript.
